# Editorial

**Published:** 1916-06

**Authors:** 


### EDITORIAL

The Atlanta Journal-Record of Medicine has its Business Office at 23 West Alabama Street where all business communications should be sent.

Remittances are payable to The Journal-Record of Medicine.

Concerning Original Communications and Editorial matters, address Richard R. Daly, M. D., 708 Empire Life Building, Atlanta, Ga.

Reprints of Original Articles will be furnished at cost price. Requests for them had best be made on the Manuscript.

Upon request, we will présent, postpaid, to each contributor of an original article, twenty copies of The Journal-Record of Medicine containing such article.

---

### INFANTILE PARALYSIS.

The nation has shown its feeling in the matter of preparedness and the rulers must follow with appropriations and some thing better than pork barrels and mending of fences. The doctors have done their part and are doing it at the camps where they are teaching prophylaxis and sanitation.

But there is another peril on its way. A highly fatal disease has already attacked the country and is daily taking its toll of the children in New York and vicinity. Infantile paralysis has become epidemic there and quarantines of various sorts are being established by the Health officers.

Children are forbidden at public assemblages of all kinds and parents are under instruction to note and give instant attention to the least appearance of departure from good health.

We do not know the period of incubation of the disease exactly. It seems to come anywhere from three to twenty days after known exposure. We do not know the disease except by its symptoms, but by putting an ailing child at rest at once and giving him such attention as we have learned is of avail, much can be done. The unhappily high death rate and the unfortunate results that often remain with the child who does not die make this the worst of chlidrens' diseases we have to encounter, and it is well to be prepared in all sections of the country against its invasion and spread.

The isolation of children suffering with even slight catarrhal conditions can do them no harm for a day or two and it may prevent the spread of the disease to others.

Let us be prepared for something of a struggle right at our doors.

---

### WHO DARES?

A thought-inducing book came to our attention and it has its notice elsewhere in the Journal. A review of it did not give opportunity for the expression of all the ideas it suggested because some of them were nowhere stated and it would be unfair to accuse an author of saying things he had not thought of or to put words into his mouth that he might eschew.

Is there some big thing back of all the misery that licentiousness and disease are causing in the world today, something that we do not talk about and yet that we know? Is there any one thing that we can easily place our finger upon that might be the prime of all the causes of sexual irregularity and yet a thing we dare not name because of tradition, because of superstition because of the opprobrium that comes parrot-like from thousands of throats in denunciation of anyone who exposes a farce hiding under the label of sacred? Is there something that can be pointed to as wrong and proved as wrong despite the reverence in which it is held, without the odium of reformer or crank or infidel being cast upon the relator? Let us see. Talking about it can not change it, for it has existed for two thousand years or more, but sometimes it is well to follow the example set forth in the early chapters of Job where everyone had his say and since the saying has been perpetuated, it was supposed to be of some value. Job suffered and suffered badly as a result of it, but his latter end was more blessed than his first which set the example for stories for future generations. This example was followed until sex stories led to problems in dramatic discussion that required the killing or the suicide of somebody and then the good old style departed. This brings us to the sex problem which was where we started and into which we shall make an excursion in fear and trembling.

What is the sex problem so far as we are concerned? Prostitution and Disease. Whatever else there may be of morals and ethics involved. If these two were eradicated, the subsidiary matters would be easily handled.

Prostitution goes a little farther back than history, for historians are fond of indicating it as being well established when their work commenced. Just why disease has accompanied it, no one can say unless those who are fond of referring everything to a definite source can attach it to Design in Creation in some manner.

That congress diseases are propagated by promiscuity is undoubted, but when and where they entered into the relationship as unwelcome and silent partners is unknown. There seems to be no reason in promiscuity, itself, for their origin. Neither can we fall back upon abiogenesis and be content with a word. We just do not know.

That disease has accompanied prostitution is evident in many historic works where its presence is unconsciously but clearly set forth. Witness the distress of a Pharaoh, of Abimelech in Genesis, of David in Psalms and the hereditary trials indicated in the early part of the Decalogue as set forth in Deut. 5. Disease and its classic symptoms were well known in those days and the indications that at the time Lot went away to a "little" city, disease was rampant behind him are fairly plain. Perhaps his ability to have wholesome children on the mountain side was set forth to show that the reason he was saved at Sodom was because of his previous escape from disease rather than to show his morality as we look upon such things nowadays.

To go back still farther we have reason to believe that the human race began in some subtropical region where the problems of existence were not complicated by having to work for food or search for clothing. In the male the instinct to procreate and the tremendously forceful desire to copulate showed themselves at the time of puberty, or before, and the males sought for gratification and fought for it. It was the natural thing. Then for some reason due to the change in climate or to migration from subtropical to temperate and ultimately to cold climates rules of conduct shaped themselves into ideas of control and then into the process of being ruled. Necessity demanded co-operation. Clothing came into use for various reasons and one of them was the covering of the female to show that she was possessed by some male. Climatic conditions also called for protection and there arose the idea of secrecy concerning the genitalia and their functions. With control there arose civilization and secrecy was its handmaiden. The possessed woman may have feared exposure because of the anger of her lord. Fear is akin to shame and the earliest records we have of the legends of sex relation tell that "they were ashamed." To account for this feeling the later writers who described this marvellous event accounted for the shame by the concept of "sin." The association of sin with the procreative anatomy was extended to its physiology and the whole matter became pregnant with the disaster that has been attracting the attention of economists during the last fifty years.

It is during that time that the cost of living has gradually increased so that the time of marriage has been postponed. In the cities, the abstemious and continent young man who wants to live decently in sexual and other ways and have a pleasant home can not see his way to getting it until about the end of the third decade. The man who does not care for these things enough to work and save for them can not get them until later. We have desire at its most intense period limited and denied by convention and at the same time the man is taught that he and his mothers and his sisters have been "born in sin." He knows that the denial of his desires is a fact. He knows that the sin of his conception is a lie. He can not fight a miserable religious tenet but let any man tell him of conceptional sin and see what happens.

A lie and a desire are bound together by civilization. He makes the most of it by getting his gratification and lying about it.

Where does he get it? From prostitutes? Well, what is a prostitute? The brothel inmate, the wanderer on the streets, the working girl who falls (and by the way that melodrama has been greatly overdrawn), or the girl who just loves a little? Where draw the lines? The part of man that knows no conscience draws no line. The young and lusty man goes forth and is touched with opportunity. She goes abroad or is met upon the steps of the vine covered porch and feels the belligerent yielding. It is the man to assault and the woman to resent and then to yield. That is really instinctive and all the education and all the pressure of 2,000 years of civilization have not changed it any more than they have altered the action of the lungs or the liver.

Right there and there only is the reason why there can not be a double standard. It is as much the part of the one to be aggressive as it is of the other to yield; it is a perfect complement and upon its fixation in even balance the integrity of the race depends.

Propinquity under the rules which would restrict desire results in sexual relations contrary to the rules. Will you keep the sexes apart?

Reforms and new rules of conduct and police regulations, all the nostra of excited fancy that look forward and never backward, have resulted in the vilest mass of prostitution and disease since history began. Ninety per cent. of the pelvic diseases of women are due to diseases of shame. Roman brothels of the Caesars can not compare with the conditions in any city today. Has anyone the courage to attack and change the rules? No. hey are sacrosanct and Rev. 22: 18-19 would be his portion.

"Born in sin"—a fetich phrase of the awful idolatry of civilization. Between the Testaments of the family Bibles in perverted. When women stop worshipping at the shrine of that insulting saint and set aside the Paulinism which makes the parlors are the records of the marriages divinely instituted and the births—black statements of foul deeds immersed in and imbued with sins that can never be eradicated, even though forgiven by divine justice! The holy union and the hellish result! No wonder the little child is lied to from the first of his intelligent questions as to whence the baby came, to the time when he learns vile things in lascivious stories as the shades of night conceal the shamed faces of listeners and raconteur. It is civilization's lie—the worst and dirtiest lie ever invented. It covers parents with shame and breeds distrust and resentment in the mind of every growing boy. It tells him that all sex control is a sham, that all mean are liars; it permits his reason to tell him to gratify his lusts and he does gratify them. Let the moralists and ethical highbrows say what they may; let each mother declare that her boy is pure, he does gratify them and the fathers know it and we doctors know it. Yea, the mothers know it for they have been girls.

When does he gratify them first? Was there ever a boy living among boys, who did not know of some boys who had gratification before puberty? It is shocking when it occurs to a little girl in one's own family; it is a sort of joke when both children are not of kin to us. Specific vaginitis begins early, so the pediatrists tell us.

Will civilization ever control sexual desire and disease? No one knows: it never has. But when people see the truth, recognize and tell it as such and thus constitute clean teaching as soon as the child begins to ask questions, we shall know. Who dares? Who dares say to the child that there is nothing sinful about the procreative act more than there is about any physiologic function, and that the reason it is surrounded by modest silence among decent people is because of its sacredness rather than its sin?

St. Paul said, 1 Cor. 7:9. "But if they cannot contain, let them marry, for it is better to marry than to burn." Paul by his own confession just before this was abnormal if not perverted. When women stop worshipping at the shrine of that insulting saint and set aside the Paulinism which makes them of use only for the quenching of burning even when married, they may be able to do better things for their children and take a higher stand in man's estimation. The children will be cleaner, both male and female. They might then be given the franchise.

All the statisticians agree that segregation and the licensing of prostitution are failures. They often point proudly to the disuse of restricted districts and then naively leave the question. Verily a little child shall lead us. Because they have placed vice beyond comparatively easy computation, there is less vice! Can anything be more farcical? We have no brief for the maintenance of the districts nor of prostitution in any form, but we do claim to be able to use a little common sense despite the essayists. We insist that we know vice and drunkenness and disease and opium smoking and bruised faces and secondary abortions and deaths from uterine toxemias even if we have not an elaborate recording system to which our eyes are glued a good share of the time. We know where we have to go on our night summons and what we find there. We know what the G. U. men are doing and what their patients are saying even when the redlights have been replaced by business houses or the "hotels and clubs" of whoredom are left to crumble in a weird and splendidly dusty loneliness. Restricted districts may not delimit vice. If a man says he knows they do not, we believe him. But neither does non-restriction, no matter what the police regulations may be. Nighthawk cabmen and chauffeurs know differently and the number of "addresses" they carry in their minds is marvellous.

Every little city has a system all its own and the open sesame in each place is merely a desire to use it. It is there because enough people to maintain it want it. They have it despite the law. Can it be cleaned up? It can in time and the method is persistent, unrelenting, complete truth telling. Who dares?

Once more, a little child must lead us and he must be told the truth when he seeks it if his virtue is to be established. When he and his generation grow up, there may be continence and control and real conformity to conventions—not otherwise.

